# The Genetic Control of the Rheumatic Heart: Closing the Genotype-Phenotype Gap

**DOI:** 10.3389/fmed.2021.611036

**Published:** 2021-03-24

**Authors:** Atiyeh M. Abdallah, Marawan Abu-Madi

**Affiliations:** Biomedical and Pharmaceutical Research Unit, Department of Biomedical Sciences, College of Health Sciences, QU Health, Qatar University, Doha, Qatar

**Keywords:** rheumatic heart, autoimmune diseases, group A streptococcus, exome sequencing, genetic association

## Abstract

Rheumatic heart disease (RHD) is a heritable inflammatory condition characterized by carditis, arthritis, and systemic disease. Although remaining neglected, the last 3 years has seen some promising advances in RHD research. Whilst it is clear that RHD can be triggered by recurrent group A streptococcal infections, the mechanisms driving clinical progression are still poorly understood. This review summarizes our current understanding of the genetics implicated in this process and the genetic determinants that predispose some people to RHD. The evidence demonstrating the importance of individual cell types and cellular states in delineating causal genetic variants is discussed, highlighting phenotype/genotype correlations where possible. Genetic fine mapping and functional studies in extreme phenotypes, together with large-scale omics studies including genomics, transcriptomics, epigenomics, and metabolomics, are expected to provide new information not only on RHD but also on the mechanisms of other autoimmune diseases and facilitate future clinical translation.

## Introduction

**Figure 1:**
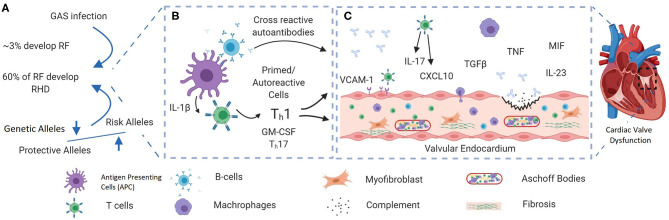
A schematic showing the possible pathogenic pathways giving rise to rheumatic heart disease (RHD) after group A streptococcal (GAS) infection. **(A)** RF/RHD is thought to be initiated by infection with rheumatogenic strains of GAS. A strong familial predisposition and the fact that only 60% of ARF patients develop RHD (1) indicate that the disease may only develop in those who are genetically predisposed. Genetic factors reported in RHD are mainly in immune response components including innate immunity genes. **(B)** Recurrent GAS infection leads to the development of autoreactive T cells and the production of cross-reactive autoantibodies. Recently, inflammasome activation has been shown to play an important role in the development of autoreactive T cells through persistent release of IL-1β (2). **(C)** Recruitment of autoreactive immune cells and cross-reactive autoantibodies to the valve interstices cause tissue damage. Recurrent and prolonged inflammation cause ongoing tissue damage, tissue fibrosis, and calcification. Figure was generated using Biorender.com.

Acute rheumatic fever (ARF) is a systemic autoimmune disease that can develop after upper respiratory tract infection with group A *Streptococcus* (GAS). Recurrent rheumatic fever and associated sustained and abnormal inflammatory responses damage the heart valves, leading to rheumatic heart disease (RHD) (Figure 1) (3). RHD remains a common cause of acquired heart disease in young adults and children in many developing countries and, to a lesser extent, in developed countries (4, 5). Globally, GAS upper respiratory tract infections are only exceeded by HIV, tuberculosis, and malaria in terms of consequent morbidity and mortality (1). In 2015, an ~33 million RHD cases worldwide were reported, leading to over 300,000 deaths and over 10 million disability-adjusted life years (4). Despite these figures, the research community and health policymakers generally neglect RHD, which received the lowest research funding relative to global disease burden over the last 5 years (6). Moreover, RHD is rarely discussed in the context of autoimmune disease in the literature.

ARF can present with several different symptoms a few weeks after a GAS episode. Rheumatic fever can be diagnosed based on the Jones criteria, developed in 1944, and then revised in 1992 and in 2015 by the American Heart Association (AHA) (Table 1) (7). ARF can be grouped into three main symptom constellations: musculoskeletal, cardiac, and neuropsychiatric. Fever is the most common presentation (>90% of patients), followed by arthritis (60–80% of patients). However, the most serious manifestation of ARF is carditis, which causes scarring and sometimes thickening of the valves, leading to permanent valvular damage that contributes to the morbidity and mortality of the disease. Some ARF patients have asymptomatic episodes, hence exposing the patients to more severe damage (8). Anywhere between 35 and 76% of ARF patients develop carditis (9, 10), predominantly left-sided, with the mitral valve affected in almost 100% of younger patients, followed by the aortic valve in 20–30% of cases and the tricuspid valve in 15–40% of cases (10). The right heart valves are nearly always affected in association with mitral or aortic disease and pulmonary valve is rarely affected. It remains unclear why particular valves are targeted in different individuals. Sydenham's chorea is the neuropsychiatric manifestation of ARF and is characterized by involuntary movements of the limbs and trunk (11). In ~20% of patients, chorea is the first disease manifestation, and while it may resolve over a few months in some patients it can last for several years, with mitral stenosis a common late complication (12).

**Table 1:** Revised Jones criteria for rheumatic fever diagnosis [adopted from (7)].

**Low risk population**	**High risk population**
**Major criteria**	
Carditis (clinical or subclinical) Arthritis – only polyarthritis Chorea Erythema marginatum Subcutaneous nodules	Carditis (clinical or subclinical) Arthritis – monoarthritis or polyarthritis Polyarthralgia Chorea Erythema marginatum Subcutaneous nodules
**Minor criteria**	
Polyarthralgia Hyperpyrexia (≥ 38.5^°^C) ESR ≥ 60 mm/h and/or CRP ≥ 3.0 mg/dl Prolonged PR interval (after taking into account the differences related to age; if there is no carditis as a major criterion)	Monoarthralgia Hyperpyrexia (≥ 38.0^°^C) ESR ≥ 30 mm/h and/or CRP ≥ 3.0 mg/dl Prolonged PR interval (after taking into account the differences related to age; if there is no carditis as a major criterion)

*ESR, erythrocyte sedimentation rate; CRP, C-reactive protein*.

The molecular mechanisms underpinning the progression of ARF to RHD are poorly understood. An autoimmune response initiated by molecular mimicry is the most commonly proposed mechanism (13), since the GAS M protein and human proteins such as cardiac myosin show structural similarities, leading to antibody cross-reactivity and cellular immune responses to human tissue (14). Cross-reactivity may not be the mechanism of progression, since autoantibodies have been detected in healthy individuals and cardiac myosin is not expressed at the cell surface (15). Approximately 60% of ARF patients develop RHD (1), and although there is a proven association between GAS infection and RHD, the triggered autoimmune process in RHD can occur autonomously after removing the stimulus (10), suggesting that after initiation of the autoimmune response via molecular mimicry, host factors, most likely genetic factors, play an important role in disease progression in susceptible individuals. This hypothesis is supported by multiple strands of evidence. First, monozygotic twins are highly concordant for the disease compared to dizygotic twins (44 vs. 12%, respectively), although these findings date to studies reported between 1933 and 1964 (16). Second, a strong familial predisposition for ARF has been noted, and the estimated heritability based on twin studies is 0.6 (95% CI 0.41–0.81), similar to the heritability of other autoimmune genetic disorders (17). Third, GAS outbreaks (18) and endemic exposure to GAS in indigenous populations (19) show that only 3–6% of individuals develop ARF, indicating that the disease may only develop in those who are genetically predisposed (20). Finally, a number of susceptibility loci have now been reported, mainly in immune-related genes and including both human leucocyte antigen (HLA) (Table 2) and non-HLA genes (35). This article summarizes the known genetic associations with RHD and discusses their role in disease pathogenesis and their potential for risk prediction and prevention.

**Table 2:** Some of the HLA class II alleles reported to be associated with ARF/RHD in different populations, illustrating the complex landscape of the disease.

**Study**	**HLA allele**	**Role: Risk (↑) or Protective (↓)**	**RF or RHD**	**Population**	**No. of participants**	**Method**	**References**
1	DQB1*0601	↑	RHD	Australia Indigenous	398 pt	GWAS	(21)
	DQA1*0301	↓			865 Ctrl		
	DRB1*0803	↑					
	DQA1*0101_DQB1*0503 (Hap)	↑					
	DQA1*0103_DQB1*0601 (Hap)	↑					
	DQA1*0301-DQB1*0402 (Hap)	↓					
2	DRB1*04-DQA1*03 (Hap)	↓	RF	Turkey	55 Pt 50 Ctrl	PCR-SSP	(22)
3	DRB1*01, DRB1*04, DRB1*07 and DQB1*02 DRB1*13	↑ (trend)↓	RHD	Turkey	100 Pt 100 Ctrl	PCR-SSP	(23)
4	DRB1*07 DRB1*11	↑↓	RF/ RHD	Turkey	173 pt 130 Ctrl	PCR-SSP	(24)
5	DQB1*08 DRB1*01	↑↓	RHD	Turkey	85 Pt 85 Ctrl	PCR-SSP	(25)
6	DRB1*15, DRB5 (DRB1*05) DRB1*04 (DRB4)	↑↓	RHD	Turkey	47 Pt 47 Ctrl	PCR-SSP	(26)
7	DRB1*07-DQA1*02 (Hap)	↑	RHD	Egypt	88 Pt 59 Ctrl	PCR-SSP	(27)
8	DRB1*0402, DRB1*1001	↑	RHD	Egypt	100 Pt 71 Ctrl	INNO-LiPA Kit	(28)
9	DRB1*07-DQB1*04 (Hap) DRB1*07-DQB1*03 (Hap) DRB1*06-DQB1*06 (Hap)	↑↑↓	RHD	Latvia	70 Pt 100 Ctrl	PCR-SSP	(29)
10	DQA1*0104 DQB1*05031	↑↑	RHD	Japan	72 pt 525 Ctrl	PCR	(30)
11	DRB1*07	↑	RHD	Pakistan	114 Pt 109 Ctrl	PCR-SSP	(31)
12	DR11 (DRB1*11) DR1 (DRB1*01)	↑↓	RHD	Uganda	96 Pt 103 Ctrl	PCR-SSP	(32)
13	DRB1*15	↑	RHD	South Indian	56 pt 254 Ctrl	PCR-SSP	(33)
14	DRB1*16-DQA1*05-DQB1*03 (Hap)	↑	RHD	Mexico	98 Pt 99 Ctrl	PCR-SSP	(34)

*RF, rheumatic fever; RHD, rheumatic heart disease; Hap, haplotype association; Pt, patients; Ctrl, control; PCR-SSP, Polymerase chain reaction sequence-specific primers. For HLA class I associations, see Martin et al. (18)*.

## Search Strategy and Selection Criteria

References for this Review were identified through searches of PubMed with the search terms “rheumatic heart,” “wide genome association,” “single nucleotide polymorphism,” and “onset” from 1990 until July, 2020. Only papers published in English were reviewed. The final reference list was generated on the basis of originality and relevance to the broad scope of this Review. We would like to stress that this is not a systematic Review.

## The Genetics of RHD

RHD is not a single gene disorder rather a complex multifactorial disease arising from the interaction between multiple genetic factors, GAS infection, and the resulting uncontrolled inflammatory response (Figure 1). Each of these genetic factors individually confers a small risk and explains a small fraction of disease heritability. Over the last few decades, genome-wide association studies (GWAS) have been the major tool used to identify genetic loci that predispose to different complex diseases. Interestingly, many of the identified susceptibility loci in RHD are clustered in key immunological pathways and are shared with other autoimmune diseases, showing evidence of natural positive selection due to their contribution to fighting infectious diseases during evolution (36–38). However, only three GWAS have been performed in RHD patients, with most other associations detected in candidate gene studies of small numbers of genes known to be functionally linked to RHD. Although important genes have been shown to be associated with RHD using the candidate-based approach, it has been difficult to successfully validate the results in different populations.

### Screening the Genome: HLA and IGH Associations

Data from the three GWASs performed in RHD patients detected associations in HLA and immunoglobulin heavy (IGH) loci (Table 3). The Pacific Islands Rheumatic Heart Disease Genetics Network performed the first GWAS in RHD in 2017 (39), which examined 1006 indigenous people in different Oceanian countries using the 300k Illumina HumanCore platform, which is considered a low-density GWAS chip. The authors justified the use of low-density GWAS by predicting that fewer variants would be needed due to linkage disequilibrium in Oceanian populations stretching over greater distances than in any other population. A novel susceptibility signal was identified in the IGH locus; the IGHV4-61^*^02 allele was associated with a 1.4-fold increased disease risk (odds ratio (OR) 1.43; 95% CI 1.27–1.61; *p* = 4.1 x 10^−9^), regarded as a small to moderate effect (41). The IGH locus is a difficult region to study, as knowledge of its polymorphisms is limited and it is poorly tagged in current arrays; indeed, only 16 genotype variants were included in this study out of the entire 1,255 kilobase locus. However, the discovery of genome-wide significance of a specific gene segment is a very important step forward to understanding the immunogenetic background of RHD.

**Table 3:** Summary of the three GWASs in different RHD populations.

	**GWAS1**	**GWAS2**	**GWAS3**
Population studied	Oceanian countries	Aboriginal Australians	South Asians & Europeans
Number of patients/control	First cohort (607 cases; 1,229 controls). Second cohort (399 cases; 617 controls)	398 RHD cases; 865 controls	First cohort (672 cases; 491 controls from South Asians). Second cohort (150 cases; 1,309 controls from UK Biobank)
Platform used	Illumina HumanCore-24 BeadChip	Illumina HumanCoreExome BeadChips.	For the first cohort: Illumina HumanCore-24 BeadChip For the second cohort: the UK Biobank Axiom Array (Affymetrix)
Identified signals	IGHV4-61 gene	HLA-DQA1 locus	HLA class III, HLA class I (HLA-B) and HLA class II (HLA-DQB1)
Role	Risk allele IGHV4-61*02	Risk haplotypes:DQA1*0101_DQB1*0503 and DQA1*0103_DQB1*0601Protective haplotype:DQA1*0301-DQB1*0402	All risk alleles
Reference	(39)	(21)	(40)

The second genome-wide association study focused on the indigenous population of Australia using the 550K Illumina Infinium HumanCoreExome platform (21). By screening 398 RHD cases, the authors identified *HLA-DQA1* (rs9272622; OR = 0.897; *p* = 1.86 × 10^−7^, for protective allele C) as the strongest association. HLA-DQB1^*^0601 (OR = 1.07; *p* = 4.06 × 10^−4^) was also identified as a risk allele, and HLA-DQA1^*^0301 (OR = 0.92; *p* = 2.71 × 10^−4^) was identified as protective. Two risk haplotypes were identified [HLA-DQA1^*^0101-DQB1^*^0503 (OR 1.44) and HLA-DQA1^*^0103-DQB1^*^0601 (OR 1.27)] and one protective haplotype [HLA-DQA1^*^0301-DQB1^*^0402 (OR 0.3)]. The study also identified HLA-DRB1^*^0803 (OR = 1.06; *p* = 0.005) as a susceptibility locus.

The third GWAS study was of a combined Indian (510 cases) and Fijian (162 cases) population, which was then confirmed in a third population from the UK (150 cases) (40). The strongest signal (rs201026476) in this study was found in the 3' untranslated region of the *PBX2* gene sited in HLA class III. The second signal was in a regulatory single nucleotide polymorphism (SNP) in *HLA-DQB1*, and the third strongest signal was in HLA class I (HLA-B). The signal in *PBX2* was believed to have a regulatory element, and the authors speculated that it may influence the expression of other HLA class III genes including complement genes such as C2 and C4. These genes and complement pathways have been proposed to play an important role in the inflammatory response in RHD patients (42). Interestingly, after conditional analysis, the HLA class III signal remained associated with RHD, indicating that the effect of rs201026476 is independent of class I and class II.

The association between RHD and HLA alleles has been reported in other small candidate marker studies. Table 2 summarizes HLA class II alleles reported to be associated with ARF/RHD in different populations. Interestingly an HLA-DQA1^*^0104 and DQB1^*^0503 association with RHD was reported in Japanese RHD patients (30). To our knowledge, HLA-DRB1^*^08 has not previously been reported, but HLA-DRB1^*^07 was reported to be a risk allele in small studies of Turkish, Pakistani, and Latvian populations (24, 29, 31). Interestingly, in an Egyptian population, the DRB1^*^07-DQA1^*^02 haplotype was associated with the mitral valve lesion subgroup of the disease (27). Other reported risk alleles include DRB1^*^01, DRB1^*^13, DRB1^*^15, and DQB1^*^08 [reviewed in (43)]. Some other HLA alleles have been reported to be protective of the disease, with examples including DRB1^*^04-DQA1^*^03 in Turkish patients and DRB1^*^06-DQB1^*^06 in Latvian patients (24, 29). In the Australian indigenous population, the HLA-DQA1^*^0301 and HLA-DQA1^*^0301-DQB1^*^0402 haplotypes were identified as protective.

These heterogeneous results demonstrate the challenge of translating genetic findings into clinically meaningful associations. Intriguingly, the genome-wide study on the indigenous Australian population did not replicate the IGHV4-61^*^02 association found in the Oceanian populations. However, both populations are indigenous, and genetic heterogeneity may have affected the results. Alternatively, the variable results may represent different exposures to different GAS strains with varying interactions with protective or risk-associated HLA alleles. Additionally, epistasis between HLA alleles and non-HLA variants may explain some of the discrepancies (44, 45). In general, the HLA and IGH associations may support the molecular mimicry hypotheses as one of the mechanisms of pathogenesis in RHD, albeit in indigenous populations.

### Candidate Genes

The candidate gene approach relies on forming biological hypotheses to predict the potential gene or locus for investigation. However, when the exact mechanism of the disease is unknown, this method may not identify its full genetic basis. Nevertheless, candidate gene case-control approaches have identified important genetic loci associated with different autoimmune diseases and have shed light on their molecular mechanisms. Relatively few candidate gene studies have been reported in RHD compared to other autoimmune diseases, and most associations remain invalidated in independent populations [reviewed in (46)]. In this section, we discuss some of these reported loci.

As post-infection autoimmune diseases involve a persistent inflammatory reaction, tumor necrosis factor-α (TNF-α) was one of the earliest genes to be studied in RHD, showing strong associations in different populations. A meta-analysis of seven studies including 735 RHD cases and 926 controls from seven different populations showed that the *TNFA-308A* allele was associated with the disease (OR 3; 95% CI 1.2–10.6; *p* = 0.02) (47). However, it is unknown whether this association is due to linkage disequilibrium (LD), the correlation between nearby variants, with HLA class I and II. HLA class II and class III are in high LD, so fine mapping of this region is important to identify causative variants. Interleukin (*IL*)*10* is another immune modulatory gene that has been studied in RHD patients from different populations and other autoimmune diseases. Three promoter polymorphisms in *IL10* (*-1082A/G, -829C/T*, and *-592C/A*) have been particularly well examined. The *IL10*-*ACC* promoter haplotype, associated with increased IL-10 production, has been shown to be a protective locus in RHD (OR = 0.6, 95% CIs 0.4–0.9; *p* = 0.01) (48). However, a meta-analysis of three different populations found that this locus shows only a non-statistically significant trend toward protection against RHD (OR = 0.70, 95% CIs 0.47–1.05; *p* = 0.08) (49). The angiotensin I-converting enzyme gene insertion/deletion polymorphism is another example of a locus showing different associations in different populations (50, 51). A recent meta-analysis of nine studies including 1333 RHD cases and 1212 controls from seven different populations showed no correlation with the disease or disease subtypes (52).

A few more genes have been linked to RHD in different studies, but there are currently no meta-analyses to confirm or reject these findings. Macrophage migration inhibitory factor (*MIF*) promoter polymorphisms *-173C* and *-794* (5-8 CATT repeats) have been associated with RHD age of onset in a Saudi Arabian population (53). The C allele at−173 was associated with higher *MIF* expression in T cell lines (54). *In vitro* and *in vivo* studies found that a greater number of CATT repeats is associated with higher gene expression (55, 56). Interestingly, these promoter polymorphisms had a dual impact on the development of RHD in the studied population. The -*173C* allele was associated with reduction in RHD risk and later disease onset, the *-794 CATT6* allele was associated with increased risk, and the *-794 CATT5* allele was associated with reduced risk. The dual influence of these alleles on other autoimmune diseases has previously been reported. In systemic lupus erythematosus (SLE), higher expression *MIF* alleles (*173C* and *794* extended alleles) were associated with a lower disease risk (57). However, lower expression alleles were associated with reduced end-stage organ involvement (58). Recently, MIF was reported to have a regulatory effect on IL-1β production via the NLRP3 inflammasome (59). Mononuclear cells derived from ARF patients peripheral blood were shown to have persistent production of IL-1β after GAS infection, which was suppressed after hydroxychloroquine treatment (2). These new findings open up new avenues for research on the role of inflammasomes in RHD and perhaps targeting the pathway therapeutically (2).

*IL17* is another immune-potent gene recently found to be associated with RHD. IL-17, also called IL-17A, is released by a subset of T helper cells known as T_h_17 cells. The *IL17* promoter polymorphism (rs2275913), which has been linked to other autoimmune diseases, was associated with RHD in patients from India (OR 2.76; *p* = 0.021). Interestingly, this association was linked to mitral valve lesions (OR 2.74; *p* = 0.039) (60). The *IL17* rs2275913-A allele was associated with higher expression of IL-17 (61). Wang et al. showed that IL-17 is needed to protect the host from GAS (62). On the other hand, prolonged and excessive T_h_17 exposure can cause valvular damage by enhancing neutrophil infiltration (63). IL-17 activates endothelial cells via the p38 MAPK pathway, which in turn induces the expression of several molecules such as VCAM and ICAM (64), which have important roles in the migration of different immune cells to the heart, particularly to the valves (65). However, the role of tissue-specific T_h_17 cells in heart and valvular tissue damage or repair has yet to be defined (66).

Another important pathway implicated in RHD is the lectin pathway, which includes mannose binding lectin-2 (*MBL2*), ficolin-1 (*FCN1*), and ficolin-2 (*FCN2*) [for a comprehensive review, see Beltrame et al. (42)]. *FCN2* promoter polymorphisms (−*986/*−*602/*−*4 GGA*) have been linked to low protein levels and an increased risk of RHD (OR 1.6, *p* = 0.02), and the AGA haplotype was protective of the disease (67). *FNC1* promoter polymorphisms *(*−*1981A, 542A*, −*144A*, and -*33T*) were associated with increased gene expression and were protective; however, two alleles *(*−*1981A* and −*144A*) increased the risk of developing valvular stenosis and mitral insufficiency (OR 0.75, *p* = 0.009 and OR 3.37, *p* = 0.027, respectively) (68). The authors proposed that alleles associated with increased production of FCN1 may help to eliminate the infection; however, prolonged inflammation under the influence of these alleles may cause tissue damage. This dual role of FCN1, similar to the dual role of MIF, highlights the complexity of the gene-environment interaction in RHD after GAS infection.

## Approaches for Determining RHD Disease Variants

Large scale omics studies including genomic, transcriptomic, epigenomic, and metabolomic approaches combined with advanced computational approaches are now providing information about the mechanisms of many infectious, autoimmune, and rheumatic diseases (69). Unfortunately, very few such studies have been performed in RHD. Nevertheless, the last two decades have seen a huge amount of “big” omics data generated in many other rheumatic autoimmune diseases (70) sharing many features with RHD such as symptoms and progression. In addition, rheumatic autoimmune diseases share many disease genetic variants, a phenomenon called pleiotropy. Therefore, the results of rheumatic autoimmune disease studies can be utilized for further investigation in RHD and may lead to potential drug repositioning (71, 72).

GWAS have successfully identified important genomic regions for rheumatic autoimmune diseases, and advances in array design and statistical approaches have significantly increased the power of GWAS. For example, several studies have used fine mapping to identify likely causative variants from GWAS using a combination of genotyping arrays with specific sets of SNPs and statistical approaches to define a smaller group of SNPs that are statistically causative. This has allowed researchers to focus on this group of SNPs, known as credible sets, for further functional studies. In a recent study, Immunochip arrays were used to identify rheumatoid arthritis (RA) risk markers in African American patients (73). However, GWAS mapping precision, that is the physical distances between the top associated variant and the causative variants, is limited, as each locus identified by GWAS is co-inherited (in LD) with other genes, regulatory elements, or non-coding transcripts. Fine mapping by imputation following genotyping arrays, where all variants are statistically inferred from the reference genome, provides a cost-effective strategy to identify many disease alleles. Wu et al. (74) showed that imputation strategies are comparable to whole genome sequencing (WGS)-based approaches for common variants but not for rare variants. This was mainly due to that fact that rare variants are not adequately presented in the reference panels such as the 1000 Genome Project, HapMap, and 1000 UK Genomes. Moreover, samples representing populations from developing countries are not sufficiently represented in these panel sets, so rare causal variants for diseases common in these countries, such as RHD, may not be present. WGS-based approaches can overcome the imputation approach limitations. In a multinational study, van Rheenen et al. identified a novel rare functional variant associated with amyotrophic lateral sclerosis using WGS-based GWAS (75). Although this approach is the current method of choice for association studies in large research centers, it is rarely applied in developing countries due to its prohibitive cost. Furthermore, it has been recommended that the usual GWAS statistical significance threshold of an observed p-value < 5 x 10^−8^ should be increased to 5 x 10^−11^ (74), which requires large numbers of participants.

Whole exome sequencing (WES) has been increasingly applied to study rare variants in different autoimmune diseases due to its low sequencing cost. For example, by dividing sarcoidosis patients into groups based on HLA markers, rare variants were detected by WES in a limited sample size, which were associated with prognosis (76). HLA subgrouping helps to reduce genetic heterogeneity. Moreover, applying WES on individuals with extreme phenotypes increases the study power by increasing the number of relevant functional variants in the study, thereby increasing the probability of detecting new associations (77). This successful identification of disease-causing variants using WES is due to the fact that the low frequency variants are enriched in exonic regions (78). However, variations in regulatory elements influence gene expression in many immune cells in autoimmune diseases (79), and the majority of confirmed disease-associated loci in autoimmunity are non-coding variants (80). It has been estimated that up to 80% of the genome is responsible for gene regulation (81). Traditional WES approaches do not cover these elements, but extended exome sequencing has been used to identify these elements (82). This approach helps to reduce costs and covers all possible important regions in autoimmune conditions. However, to really understand how these regulatory elements contribute to autoimmune diseases, we need to understand how these elements regulate gene function at the cellular level (83).

Once a regulatory element has been associated with a complex disease, follow-up experiments are often conducted to understand the mechanism by which the variants influence phenotype. As an initial step, researchers carry out gene expression and DNA methylation and histone modification studies in various cell types. Genetic variants that influence gene expression level are called expression quantitative trait loci (eQTLs). eQTLs are classified according to their location (local or distant) or mode of action (*cis* or *trans*) [for a review, see (84)]. eQTLs are influenced by the environment, and some eQTLs behave differently under different conditions (85). Therefore, an important aspect of eQTL studies is to correlate the genetic locus with the correct cell type in the correct activation state in both health and disease. Although cell separation techniques may affect cellular status, they have provided relevant information regarding gene expression (known as transcript abundance) in many cases (86, 87). An interesting new approach is single-cell eQTL and the establishment of the Single-cell eQTLGen Consortium (sc-eQTLGen), which aims to map the upstream and downstream interactions of disease-related eQTLs in individual immune cell types (88). Unfortunately, in RHD, the immune cells most relevant to its pathogenesis remain unknown. In a recent study, Kim et al. reported disruption in the IL-1β granulocyte-macrophage colony-stimulating factor (GM-CSF) cytokine pathway in peripheral blood mononuclear cells from rheumatic fever patients stimulated with heat-inactivated GAS strains and studied gene expression in these cells (89). They identified hydroxychloroquine as an adjunctive therapy that might limit the dysregulation of this pathway. Further work to identify disease-causing pathways using specific cell populations might provide accurate insights into disease pathogenesis and correlations between gene expression and genetic variants.

## Final Remarks and Conclusions

In the COVID-19 era, it is becoming even more important to identify pleiotropic immune mechanisms in rheumatic diseases since there may be shared mechanisms and pathways that are exploitable from the therapeutic perspective (90). The environmental trigger for autoimmunity in RHD is known; however, the inflammatory processes that occur after the infection and lead to RHD are still not well-understood. Understanding these processes at the cellular and molecular levels is crucial to deciphering RHD pathogenesis. In the last 3 years, while we have seen some advances in RHD genetics, GWAS studies have provided inconsistent results. One explanation is that these studies were mainly carried out in indigenous populations and trans-ethnic differences influence disease mechanisms. Another important factor is the widespread diversity of GAS strains, which may evoke different immune signaling pathways. Establishing which cytokine and cell signaling pathways are dysregulated in specific cell types in a specific ethnic population and the identification of the genetic variants that cause these processes are required to gain further insights into the immunological mechanisms of RHD. Exome sequencing and fine mapping will produce robust results. However, the lack of reference control data from different developing countries will challenge the identification of rare variants. Population genome project initiatives in developing countries and the Middle East, such as Qatar Genome (91), are expected to provide good reference data to help with these efforts.

Future studies should focus on understanding the function of any discovered variant through transcriptomic, epigenomics, proteomics, and metabolomics studies. Transcriptomic studies will define new disease pathways and pathogenic cell types, while epigenomic studies will link genetic promoters and enhancers with target genes. In rheumatic diseases, different cells play different roles; therefore, it is important to identify pathologically important immune cells. Single-cell technologies combined with advanced computational approaches and/or machine learning developed over the last decade have enabled integrative analysis of single-cell multi-omics data in different immunological diseases (92, 93). Future RHD studies will require collaboration between wet lab scientists, clinicians, and computational biologists to validate associations. Moreover, sequencing individuals with extreme phenotypes such as early disease onset will help to detect the strongest and most precise genetic associations. An important lesson emerging from other rheumatic diseases is that genetic associations influence disease phenotype and not only generic disease susceptibility. Therefore, detailed and accurate clinical phenotyping would also be helpful to identify new genetic factors that may influence disease progression and the underlying mechanisms. Pursuing this course will open up new avenues for early diagnosis, new therapeutic targets, and vaccine development.

## Author Contributions

AA wrote the first draft of the manuscript and drafted the figure. MA-M edited and revised the manuscript and contributed figures. All authors revised the manuscript critically and approved the final version of the manuscript.

## Conflict of Interest

The authors declare that the research was conducted in the absence of any commercial or financial relationships that could be construed as a potential conflict of interest.
